# Epitope-specific antibody fragments block aggregation of AGelD187N, an aberrant peptide in gelsolin amyloidosis

**DOI:** 10.1016/j.jbc.2024.107507

**Published:** 2024-06-27

**Authors:** Laura Leimu, Patrik Holm, Anna Gąciarz, Oskar Haavisto, Stuart Prince, Ullamari Pesonen, Tuomas Huovinen, Urpo Lamminmäki

**Affiliations:** 1 R&D, Orion Pharma, Orion Corporation, Turku, Finland; 2 Faculty of Medicine, Institute of Biomedicine, University of Turku, Turku, Finland; 3 Department of Life Technologies, University of Turku, Turku, Finland; 4 Organon R&D Finland, Turku, Finland; 5 Mobidiag, A Hologic Company, Espoo, Finland; 6 MediCity Research Laboratory, University of Turku, Turku, Finland

**Keywords:** protein misfolding, gelsolin amyloidosis (AGel amyloidosis), amyloid, drug discovery, aggregation inhibition, antibody, antibody engineering, phage display

## Abstract

Aggregation of aberrant fragment of plasma gelsolin, AGelD187N, is a crucial event underlying the pathophysiology of Finnish gelsolin amyloidosis, an inherited form of systemic amyloidosis. The amyloidogenic gelsolin fragment AGelD187N does not play any physiological role in the body, unlike most aggregating proteins related to other protein misfolding diseases. However, no therapeutic agents that specifically and effectively target and neutralize AGelD187N exist. We used phage display technology to identify novel single-chain variable fragments that bind to different epitopes in the monomeric AGelD187N that were further maturated by variable domain shuffling and converted to antigen-binding fragment (Fab) antibodies. The generated antibody fragments had nanomolar binding affinity for full-length AGelD187N, as evaluated by biolayer interferometry. Importantly, all four Fabs selected for functional studies efficiently inhibited the amyloid formation of full-length AGelD187N as examined by thioflavin fluorescence assay and transmission electron microscopy. Two Fabs, neither of which bound to the previously proposed fibril-forming region of AGelD187N, completely blocked the amyloid formation of AGelD187N. Moreover, no small soluble aggregates, which are considered pathogenic species in protein misfolding diseases, were formed after successful inhibition of amyloid formation by the most promising aggregation inhibitor, as investigated by size-exclusion chromatography combined with multiangle light scattering. We conclude that all regions of the full-length AGelD187N are important in modulating its assembly into fibrils and that the discovered epitope-specific anti-AGelD187N antibody fragments provide a promising starting point for a disease-modifying therapy for gelsolin amyloidosis, which is currently lacking.

Gelsolin amyloidosis (AGel amyloidosis) is a hereditary systemic amyloidosis in which, a point mutation in the gelsolin gene leads to the formation of aggregating fragments of plasma gelsolin. The best-known variant of the disease is the Finnish variant, where the mutation causes the aspartic acid at position 187 of the gelsolin protein to be substituted with an asparagine (D187N mutation) (1). Two subsequent proteolytic events, intracellular furin cleavage (2) and extracellular membrane-type 1 matrix metalloproteinase cleavage (3), result in the formation of intrinsically disordered amyloidogenic peptides. The main product is an 8-kDa fragment comprising 70 amino acid residues, AGelD187N 173 to 242 (later AGelD187N). Finnish gelsolin amyloidosis causes a wide variety of ophthalmological, neurological, cutaneous, and oral symptoms that together with repeated surgeries cause a clinically significant disease burden (4). As for most amyloid diseases, there is only symptomatic treatment available for gelsolin amyloidosis, and a disease-modifying treatment would be highly desirable.

A nanobody-based approach has been studied by Gettemans *et al.* to delay the progression of gelsolin amyloidosis (5, 6, 7). In their latest study, a bispecific nanobody was developed that was able to simultaneously shield mutant plasma gelsolin from intracellular furin and extracellular membrane-type 1 matrix metalloproteinase activity (7). A significant decrease in AGel amyloid buildup was observed both in muscle and heart tissue in a transgenic AGel mouse model. However, this approach required intracellular delivery of protective nanobodies *via* adeno-associated virus serotype 9 (AAV9). Gene therapy would be an elegant way to deal with a monogenic disorder such as gelsolin amyloidosis, but the technology is not yet fully established (8). Another promising approach to modify disease progression is to inhibit the formation of harmful aggregates by targeting the aberrant amyloidogenic peptide AGelD187N. This strategy has been attempted with small molecules, either alone or conjugated to nanoparticles, and with peptides (9, 10, 11, 12). The investigated small molecules are known bioactive compounds, usually known modulators of some other amyloidogenic proteins like amyloid-β (9, 10, 12, 13, 14). In a recent structure-based approach, Bollati *et al.* designed three novel peptides resembling a short sequence in AGelD187N that is believed to represent the amyloidogenic core of the fragment (11). Despite promising aggregation inhibition data *in vitro*, limited binding data of suggested inhibitors to AGelD187N is reported. The only reported binding constants indicate weak binding (10). This is in line with earlier literature (9, 15, 16, 17, 18), but it raises a concern about the true selectivity of the suggested inhibitors to AGelD187N and their actual effectiveness in an *in vivo* setting.

In contrast to small molecules and peptides, antibodies can bind to disordered proteins or their aggregates with high affinity (19). Recently, two antibodies directed against amyloid-β, aducanumab and lecanemab, have been approved for the treatment of Alzheimer's disease. However, the clinical success of anti-amyloid immunotherapies against neurodegenerative diseases has been modest despite decades of intensive research (20, 21, 22, 23). This is due to several challenges. First, misfolded proteins in the brain are difficult to target with antibodies because they are behind the blood-brain barrier and mostly intracellular. Second, neurodegenerative diseases have a multifactorial etiology and diverse disease course. For systemic amyloidoses, according to our knowledge, there are currently several anti-amyloid immunotherapies under development (24, 25). From these, transthyretin amyloidosis (ATTR amyloidosis) has a hereditary form of the disease (ATTRv) with known pathogenetic mutations, but still, the disease diversity complicates obtaining proof of the effective treatments (26). In contrast, Finnish gelsolin amyloidosis has a straightforward disease pathophysiology with 100% penetrance and early onset of disease (27). Another feature differentiating gelsolin amyloidosis from other protein misfolding diseases is that the amyloidogenic AGelD187N monomer does not have any physiological role in the body, making it a suitable therapeutic target for neutralization.

In this study, we report high-affinity antigen-binding fragment (Fab) antibody fragments against the monomeric gelsolin amyloidogenic peptide AGelD187N, which is aberrantly formed in the Finnish variant of gelsolin amyloidosis. The antibody fragments were generated by phage display using multiple peptide antigens and antibody light chain variable domain and antibody heavy chain variable domain (VL/VH) shuffling. We showed that the developed Fabs binding to different epitopes efficiently inhibited amyloid formation as monitored by thioflavin T (ThT) fluorescence and electron microscopy. We also confirmed by size-exclusion chromatography (SEC) combined with multiangle light scattering (SEC-MALS) that no small, possibly non-ThT binding, aggregates were formed after successful inhibition of amyloid formation by the most promising aggregation inhibitor, Fab 4.

## Results

### Generation of epitope-specific Fab antibody fragments that bind to AGelD187N

#### Discovery strategy

**Figure 1:**
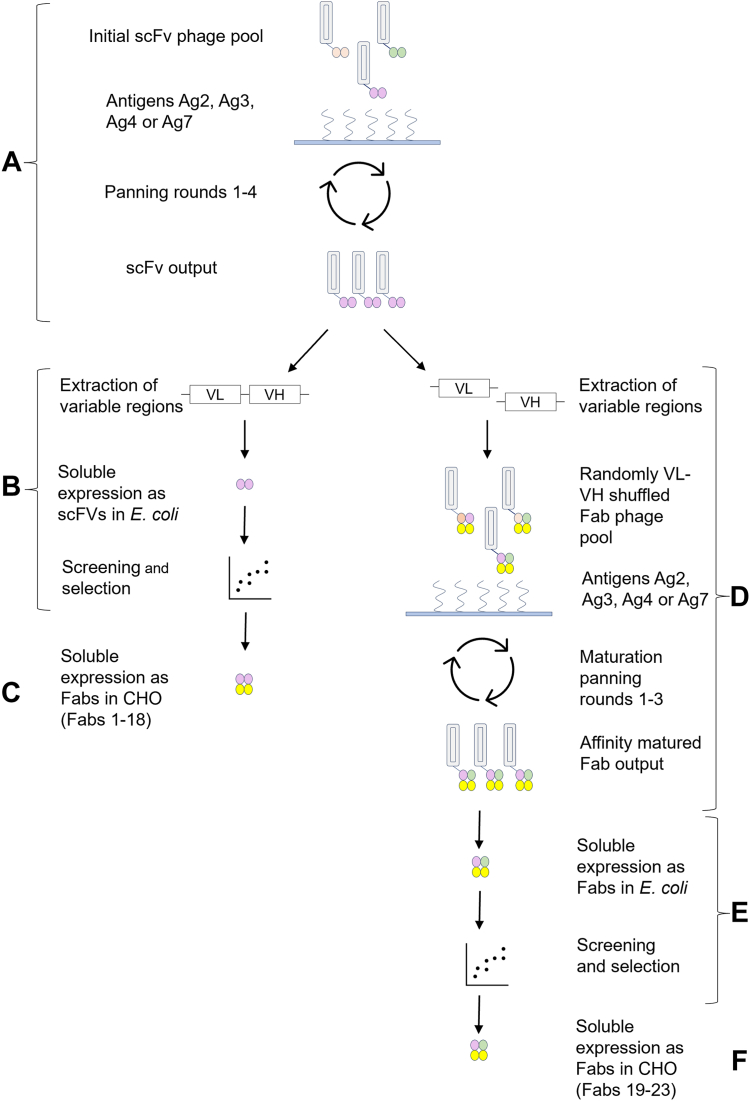
**Schematic overview of the phage display strategy leading to Fabs 1 to 23.***A*, the initial scFv phage pool was panned separately against all antigens until significant enrichment was observed, as determined by the number of colonies on output plates and phage immunoreactivity. *B*, VL-VH regions of selected phage pools were extracted and subcloned into an expression vector for soluble scFv. Single scFv clones were expressed in *E. coli*, screened, and characterized. *C*, selected clones were converted to Fabs, expressed in CHO cells, and characterized. *D*, alternatively, the VL and VH regions from selected scFv outputs were extracted as pools and recombined with human CL and CH1 domains to randomly generate VL/VH-shuffled Fab genes. These genes were subcloned back into the phagemid. Three rounds of panning were performed to select high-affinity binders. *E*, variable regions of selected phage pools were extracted and subcloned into an expression vector for expression in *E. coli* and screening of soluble, monoclonal Fabs. *F*, selected clones were expressed in CHO cells and characterized. CHO, Chinese hamster ovary; Fab, antigen-binding fragment; scFv, single-chain variable fragment; VH, antibody heavy chain variable; VL, antibody light chain variable domain.

**Figure 2:**
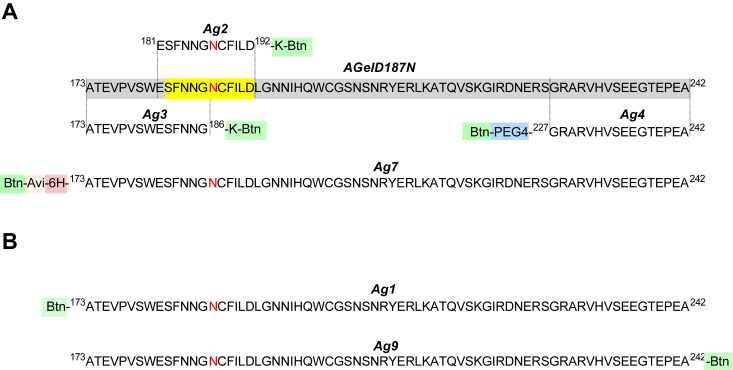
**AGelD187N and the antigens used in the study.***A*, AGelD187N and antigens Ag2, Ag3, Ag4, and Ag7 that were used for biopanning. The full-length AGelD187N (*gray*), its proposed fibril-forming region (*yellow*), and the mutation site in residue 187 (*red* N). Amino acid numbering is based on UniProt entry P06396-1, human plasma gelsolin, without the signal peptide 1 to 27. Antigen sequences are in alignment with AGelD187N. Ag 2 and Ag3 have the C-terminal lysine biotinylated at the epsilon-amino group (-K-Btn, *green*), and Ag4 has a biotinylated (Btn-, *green*) PEG4 linker (PEG4-, *blue*) in the amino terminus. Other antigens were produced by chemical synthesis, but Ag7 is a product of recombinant bacterial expression and BirA enzyme-mediated *in vivo* biotinylation. It has a biotin tag (Btn-, *green*) followed by an Avi tag (Avi-, *orange*) and a hexahistidine tag (6H-, *light red*) in the amino terminus. *B*, antigens Ag1 and Ag9 that were used for affinity determination. Ag1 has aminoterminal biotinylation (Btn-, *green*) and Ag9 has C-terminal biotinylation (-Btn, *green*).

We used single-chain variable fragment (scFv) phage display and multiple peptide antigens to generate scFvs that were subsequently converted into Fab format both individually, and as a library with inherent VL/VH shuffling, to generate epitope-specific Fab antibodies to AGelD187N, as described in Figure 1. The amino acid sequence of AGelD187N and all antigens used with their biotin tags are presented in Figure 2.

#### Generation of epitope-specific scFv phage pools

The initial scFv phage pool was an equal mixture of two closely related synthetic scFv phage libraries denoted scFvP (28) and scFvM (29) constructed in the same human framework (Fig 1*A*). We generated enriched scFv phage pools binding to specific epitopes of AGelD187N by panning scFv phage libraries against peptides that corresponded to three specific regions of AGelD187N (Fig. 2*A*). These synthetic peptides represented the proposed fibril-forming region (Ag2), the N-terminal region (Ag3), and the C-terminal region (Ag4) of AGelD187N. In addition, the scFv phage libraries were panned against the full-length recombinant AGelD187N (Ag7). The first two or three panning rounds were performed using a solid-phase protocol, where avidin-coated magnetic beads were loaded with an excess of biotinylated antigens before incubation with phage. For the third and fourth or only for the fourth final panning round, a solution-phase protocol was used. In the solution-phase protocol, avidin beads were used to capture phage-antigen complexes formed upon preincubation. Three concentrations of antigens were used in the preincubation to find conditions for the capture of high-affinity binders. Specificities of scFv phage pools were assessed after each round using phage immunoassays. Signal-to-background ratios ranging from 140 to 1611 were achieved after three or four rounds of panning. The detailed panning conditions and results for the third and fourth panning rounds are listed in Table S1.

#### Isolation of monoclonal scFv and Fab antibody fragments

The results above motivated us to isolate and investigate the binding profiles of single scFv clones (Fig. 1, *B* and *C*). In total, 744 scFv single clones were expressed in *Escherichia coli* in a soluble screening format, of which 482 showed higher than cutoff binding (signal-to-background ratio >5) to respective panning antigen in an immunoassay on cell lysates. A confirmatory secondary screening of affinity-purified clones and sequence redundancy analysis narrowed the selection to 18 clones of interest. Representative clones were reexpressed in *E. coli* and ranked based on their equilibrium dissociation constant, K_D_, measured by biolayer interferometry. In addition, epitope specificities were determined by pairwise epitope binning experiments. Clones showing nM range affinity and unique binding epitopes are presented in Table S2. These promising results prompted us to pursue our research on conversion to the Fab format. The variable regions of the 18 scFvs of interest were assembled as Fab genes together with the constant domain of the human kappa light chain (hCL_Κ_) and the constant domain 1 of the human immunoglobulin G1 (IgG1) heavy chain (hIgG1-CH_1_) into mammalian expression vectors, followed by expression in Chinese hamster ovary (CHO) cells and purification using affinity and SEC.

#### Generation of epitope-specific VL/VH-shuffled Fab phage pools and isolation of monoclonal Fab antibody fragments

Furthermore, to identify novel VL-VH combinations with higher affinity, variable regions from the third and fourth round scFv phage output libraries were cloned by PCR, assembled to Fab fragments, and cloned into the phagemid vector for further rounds of phage display selection (Fig. 1, *D*–*F*). In addition to converting the antibodies from scFv to a more stable Fab scaffold, the method randomly shuffled VL and VH domains between the clones. These VL/VH shuffled libraries were panned for three rounds against the same antigens as described for scFvs and specificities of Fab phage pools were assessed using phage immunoassays. Results from the third panning round are presented in Table S3. Screening of individual Fab clones yielded active binders for all panning antigens. Based on their binding properties, five VL/VH-shuffled Fabs were expressed in CHO cells and purified.

#### Selected Fabs

Overall, 23 clones were expressed as Fabs 1 to 23 in CHO cells. Expression levels and correct molecular weights of Fabs were assessed by SDS-PAGE (Fig. S1). The binding affinities to the full-length AGelD187N were determined by biolayer interferometry using two antigens both corresponding to the complete amino acid sequence of AGelD187N (Fig. 2*B*); one of them had aminoterminal biotinylation (Ag1) and the other one C-terminal biotinylation (Ag9). The unique amino acid sequence of the complementarity-determining region of each Fab clone and the binding affinity to both antigens are shown in Table 1. After conversion from scFv to Fab, 11 of the 18 directly converted Fabs (method a) retained their binding ability. VL/VH shuffling (method b) yielded clones with over 10-fold higher binding affinity for Ag2 and Ag3 compared to direct conversion. For the ten highest affinity Fabs, the binding affinities determined with a single concentration (300 nM) were confirmed using the kinetic titration series method (30) and six concentrations (31 nM–1 μM). These results were in line with the results achieved with one concentration (Table S4). Based on binding affinity and antigen specificity, Fabs 4 (anti-Ag4 selection), 14 (anti-Ag7 selection), 19 (anti-Ag3 selection), and 21 (anti-Ag2 selection) were selected for aggregation inhibition studies. Among these, Fabs 4 and 14 were directly cloned from scFv to Fab, and Fabs 19 and 21 were isolated from VL/VH-shuffled Fab phage display libraries.

**Table 1:** Amino acid sequences of CDRs and binding affinities of the selected Fab clones

			CDR sequences^a^						Affinity, K_D_(nM)^b^			
			Light chain			Heavy chain			Ag1 (N-Btn)^c^		Ag9 (C-Btn)^d^	
Fab clone	Method^e^	Antigen^f^	L1	L2	L3	H1	H2	H3	Mean	SD	Mean	SD
**Fab 19**	**b**	**Ag3**	**SQSVSSSYLN**	**YGASSRATGV**	**QQGYSSPP**	**GFTFSSYWMS**	**SISSSGGSTH**	**ARVLGDY**	**-**	**-**	**120**	**21**
Fab 16	a	Ag3	SQSVSSSYLN	YGASSRATGV	QQGNYSPL	GFTFSSYLMS	SINPSGGSTY	AKRAFDI	-	-	4770	156
Fab 18	a	Ag3	SQSVSSSYVS	YGASSRATGV	LQSKYNPH	GFTFSSYSMH	SITPSGGSTH	ATYNY	-	-	2650	205
Fab 17	a	Ag3	SQSVSSSNLS	YGASSRATGV	LQSYYNPH	GFTSPGYSMH	SIASSGGSTY	TRYAY	-	-	3900	2180
**Fab 21**	**b**	**Ag2**	**SQSVSSSYLN**	**YGASSRATGV**	**LQGNYNPP**	**GFTFSSYLMD**	**RIDPSGGSTY**	**VLGGFYY**	**125**	**2**	**127**	**8**
Fab 1	a	Ag2	SQSVSSSYLN	YGASSRATGV	QQNYYSPH	GFTFSSYAMN	GINPSGGSTN	VTPPNL	1580	643	2500	523
Fab 9	a	Ag2	SQSVSSSYLA	YGASSRATGV	LQDYSTPY	GFTFSSYGMH	QITPSGGSTH	ARWYDY	-	-	-	-
Fab 8	a	Ag2	SQSVSSSYLN	YGASSRATGV	LQGNYNP	GFTFSSYLMD	RIAPSGGSTY	ASGGFGY	-	-	-	-
Fab 10	a	Ag2	SQSVSSSSLN	YGASSRATGV	HQGNYNPR	GFTFSSYSMN	QITPSGGSTY	ARPDGDY	-	-	-	-
Fab 5	a	Ag2	SQSVSSSSLN	YGASSRATGV	QQGYSSPP	GFTFSSYWMS	SISSSGGSTH	ARVLGDY	-	-	344	6
**Fab 4**	**a**	**Ag4**	**SQSVSSSSLN**	**YGASSRATGV**	**QQHTYDPP**	**GFTFSSYWMN**	**EINPSGGSTH**	**ASDAFDY**	**33**	**7**	**46**	**2**
Fab 20^g^	b	Ag4	SQSVSSSSLN	YGASSRATGV	QQHTYDPP	GFTFSSYWMN	EINPSGGSTH	ASDAFDY	37	5	54	8
Fab 23	b	Ag4	SQSVSSSSLG	YGASSRATGV	QQHTYDPP	GFTFSSYLMD	RIDPSGGSTY	VLGGFYY	41	9	57	11
Fab 13	a	Ag4	SQSVSSSYLN	YGASSRATGV	QQANYNPL	GFTFSSYWMN	EINPSGGSTD	ATDAFDY	54	5	83	2
Fab 15	a	Ag4	SQSVSSSYLN	YGASSRATGV	QDYYVPF	GFTFSSYWMD	GIAPSGGSTN	ARYGYYGFGD	97	16	94	15
Fab 3	a	Ag4	SQSVSSSYLD	YGASSRATGV	QQRTYYPF	GFTFSSYWME	EINPSGGSTH	VRPQYQWDDV	101	33	120	33
**Fab 14**	**a**	**Ag7**	**SQSVSSSNLN**	**YGASSRATGV**	**LQNNYAPR**	**GFTFSSYLMT**	**GIAPSGGSTY**	**ARPRWRRDASDY**	**76**	**10**	**92**	**4**
Fab 22	b	Ag7	SQSVSSSYLN	YGASSRATGV	QQDSYIPP	GFTFSSYWMN	EINPSGGSTH	ASDAFDY	63	16	87	21
Fab 7	a	Ag7	SQSVSSSSLN	YGASSRATGV	HQRYYNPR	GFTFSSYLMT	SITPSGGSTN	AKPARWSAV	868	385	770	252
Fab 6	a	Ag7	SQSVSSSYLN	YGASSRATGV	LQRSSSPY	GFTFSSYLMT	SISPSGGSTN	ARPRYRVAPSY	-	-	-	-
Fab 12	a	Ag7	SQSVSSSNLN	YGASSRATGV	QQHNSDPP	GFTFSSYVMT	AIAPSGGSTY	GFARPSRGTVVSY	-	-	-	-
Fab 2	a	Ag7	SQSVSSSYLN	YGASSRATGV	LQDYSIPH	GFTFSSYLMH	WISPSGGSTD	VRQGSYRSDY	-	-	-	-
Fab 11	a	Ag7	SQSVSSSYLG	YGASSRATGV	LQRNYIPH	GFTFSSYLMT	SITPSGGSTD	ARPQIGRYRSV	-	-	-	-

The clones are grouped according to the respective selection antigens used in biopanning.

CDR, complementarity-determining region; SD, standard deviation. = not detected. **Fabs in bold font** were selected for the aggregation inhibition experiments.

a CDR sequences follow the international ImMunoGeneTics information system (IMGT) definitions.

b K_D_ values were determined using biolayer interferometry (kinetic screening method).

c Ag1 is synthetic full-length AGelD187N with aminoterminal biotinylation.

d Ag9 is synthetic full-length AGelD187N with C-terminal biotinylation.

e (a) Fab was generated *via* direct conversion from scFv. (b) Fab was isolated from a Fab library generated from enriched anti-AGelD187N scFv outputs after biopanning.

f Antigen used for selection in biopanning.

g Fab 20 has leucine in position L:198 (outside CDR region), whereas Fab 4 has proline.

### Inhibition of AGelD187N amyloid formation

To evaluate the functional activity of selected Fabs, we used our previously established *in vitro* aggregation assay that monitors the full-length AGelD187N 173 to 242 seed-induced aggregation by ThT fluorescence (31). Preformed fibrils were used to bypass the rate-limiting nucleation step in the aggregation process and initiate the rapid fibril growth phase simultaneously in all wells. To assess whether Fabs 4, 14, 19, and 21 can interfere with the amyloid formation of AGelD187N, they were tested at 2.5 μM, 5 μM, and 10 μM concentrations representing 1:0.25, 1:0.5, and 1:1 AGelD187N to Fab stoichiometries in the aggregation assay. In addition to examining the kinetic aggregation curves, the functional activity was evaluated using two different parameters: the effect of Fabs on the AGelD187N aggregation rate and the maximum ThT fluorescence intensities. The aggregation rates were calculated by fitting a straight line to the linear range of 0 to 110 min of each aggregation curve by nonlinear regression. Maximum ThT fluorescence intensities were used to evaluate the number of amyloids formed. After the assay, the effects were validated using transmission electron microscopy (TEM).

**Figure 3:**
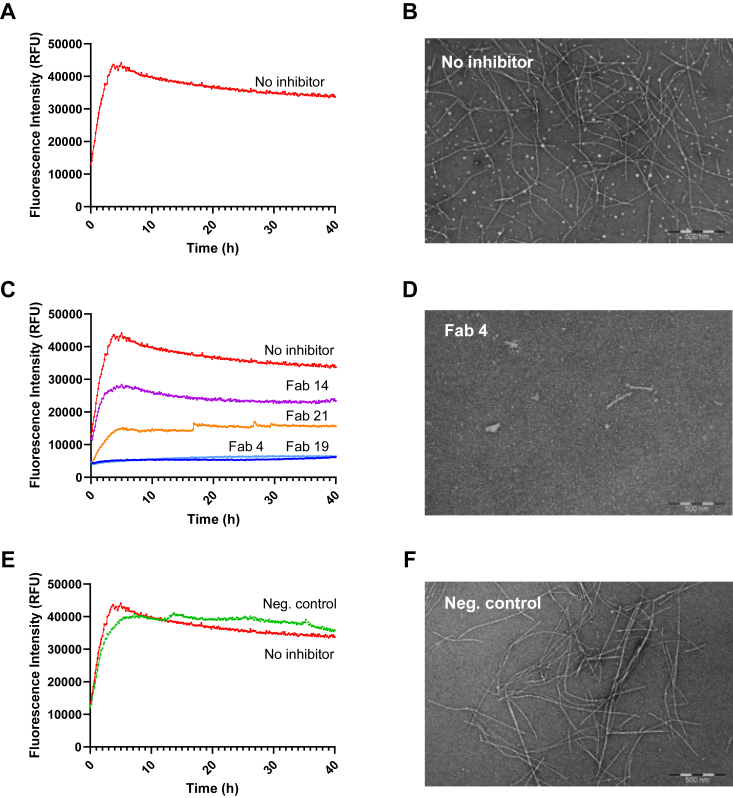
**Inhibition of AGelD187N aggregation by selected anti-AGelD187N Fabs.***A*, amyloid formation kinetics in the absence of Fabs monitored continuously for 40 h by ThT fluorescence. The mean curve of three replicate measurements is shown. Individual kinetic traces are shown in Fig. S2*A*. *B*, representative electron micrograph after the aggregation assay performed in the absence of Fabs. The scale bar represents 500 nm. *C*, amyloid formation kinetics in the presence of 10 μM Fab 4 (*blue*), Fab 19 (*light blue*), Fab 21 (*orange*), and Fab 14 (*purple*). For each Fab, the mean curve of three replicate measurements is shown. Individual kinetic traces are shown are shown in Fig. S2, *B*–*E*. *D*, representative electron micrograph after the aggregation assay performed in the presence of 10 μM Fab 4. The scale bar represents 500 nm. Corresponding images for Fab 19, Fab 21, and Fab 14 are shown in Fig. S3. *E*, amyloid formation kinetics in the presence of 10 μM negative control Fab (*green*). The mean curve of three replicate measurements is shown. Individual kinetic traces are shown in Fig. S2*F*. *F*, representative electron micrograph after the aggregation assay performed in the presence of 10 μM negative control Fab. The scale bar represents 500 nm. Fab, antigen-binding fragment; ThT, thioflavin T.

At 10 μM concentration, all tested Fabs inhibited amyloid formation effectively (Figs. 3*C* and S2). Fab 4, which binds to the C-terminal region of AGelD187N, and Fab 19, which binds to the N-terminal region of AGelD187N, both arrested the amyloid formation fully. In the TEM images, there were only a few short (<0.5 μm) fibrils present in Fab 4 (Fig. 3*D*) and Fab 19 samples (Fig. S3*A*). Fab 21, which binds to the proposed fibril-forming region of AGelD187N, caused the fluorescence intensity to decrease by over 50%, and Fab 14, which was panned against full-length AGelD187N and therefore its binding region is unknown, caused the fluorescence intensity to decrease by over 30%. The decrease in amyloid quantity was also clearly visible in the TEM images (Fig. S3, *B* and *C*).

**Figure 4:**
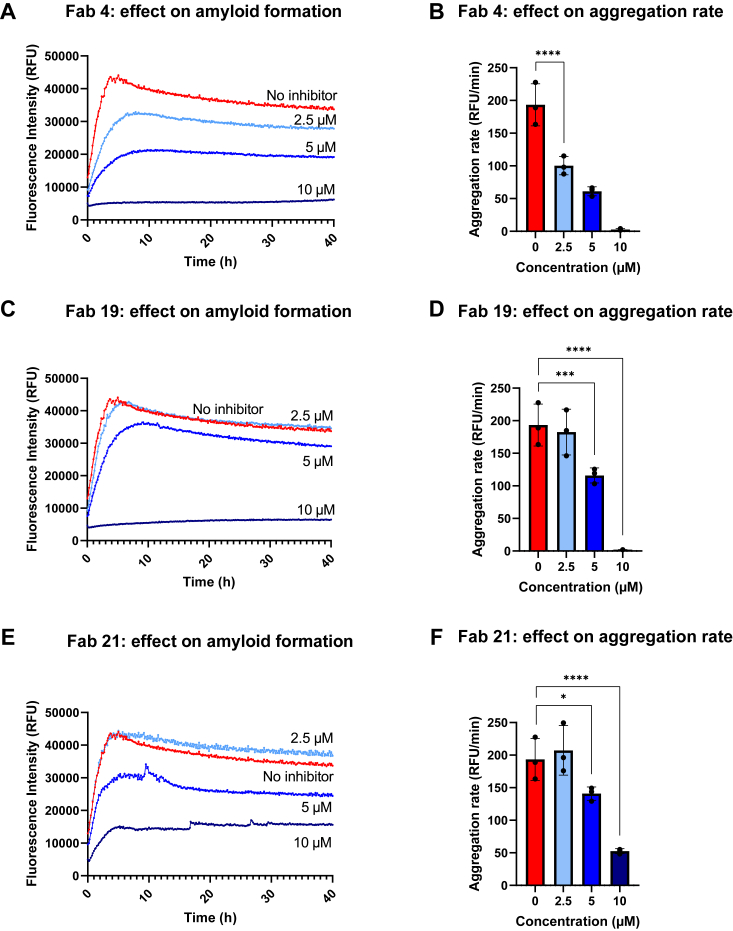
**Fab 4, Fab 19, and Fab 21 inhibit AGelD187N amyloid formation in a concentration-dependent manner.***A*, amyloid formation in the presence of 0 μM, 2.5 μM, 5 μM, and 10 μM Fab 4 monitored continuously for 40 h by ThT fluorescence. The mean curve of three replicate measurements is shown. *B*, aggregation rate in the presence of 0 μM, 2.5 μM, 5 μM, and 10 μM Fab 4. The aggregation rates were calculated by fitting a straight line to the linear range of 0 to 110 min of each aggregation reaction by nonlinear regression. The data are plotted as mean ± standard deviation (SD). The individual data points superimposed on each bar represent three replicate measurements. *C* and *D*, amyloid formation and aggregation rate in the presence of 0 μM, 2.5 μM, 5 μM, and 10 μM Fab 19. *E* and *F*, amyloid formation and aggregation rate in the presence of 0 μM, 2.5 μM, 5 μM, and 10 μM Fab 21. ∗∗∗∗*p* < 0.0001; ∗∗∗*p* = 0.0004; ∗*p* = 0.0239; (one-way ANOVA test, followed by Dunnett's multiple comparisons test). Fab, antigen-binding fragment; ThT, thioflavin T.

Fabs 4, 19, and 21 showed concentration-dependent inhibition of amyloid formation (Fig. 4). Both the aggregation rates (Fig. 4, *B*, *D* and *F*) and the maximum ThT fluorescence intensities (Fig. S4, *A*–*C*) decreased in a concentration-dependent manner. Fab 4 was the most potent aggregation inhibitor as it had a significant effect on both the aggregation rate (Fig. 4*B*) and maximum signal (Fig. S4*A*) even at the lowest 2.5 μM concentration. Fabs 19 and 21 had significant effects on the aggregation rate at 5 μM concentration, with the effect of Fab 19 being greater than that of Fab 21 (Fig. 4, *D* and *F*). At 2.5 μM concentration, these two Fabs did not show significant effects compared with the aggregation process of AGelD187N without inhibitor. The fourth investigated clone, Fab 14, which showed aggregation inhibition at the highest concentration, surprisingly seemed to induce aggregation at lower concentrations (Fig. S5).

### Inhibition of soluble AGelD187N aggregates

**Figure 5:**
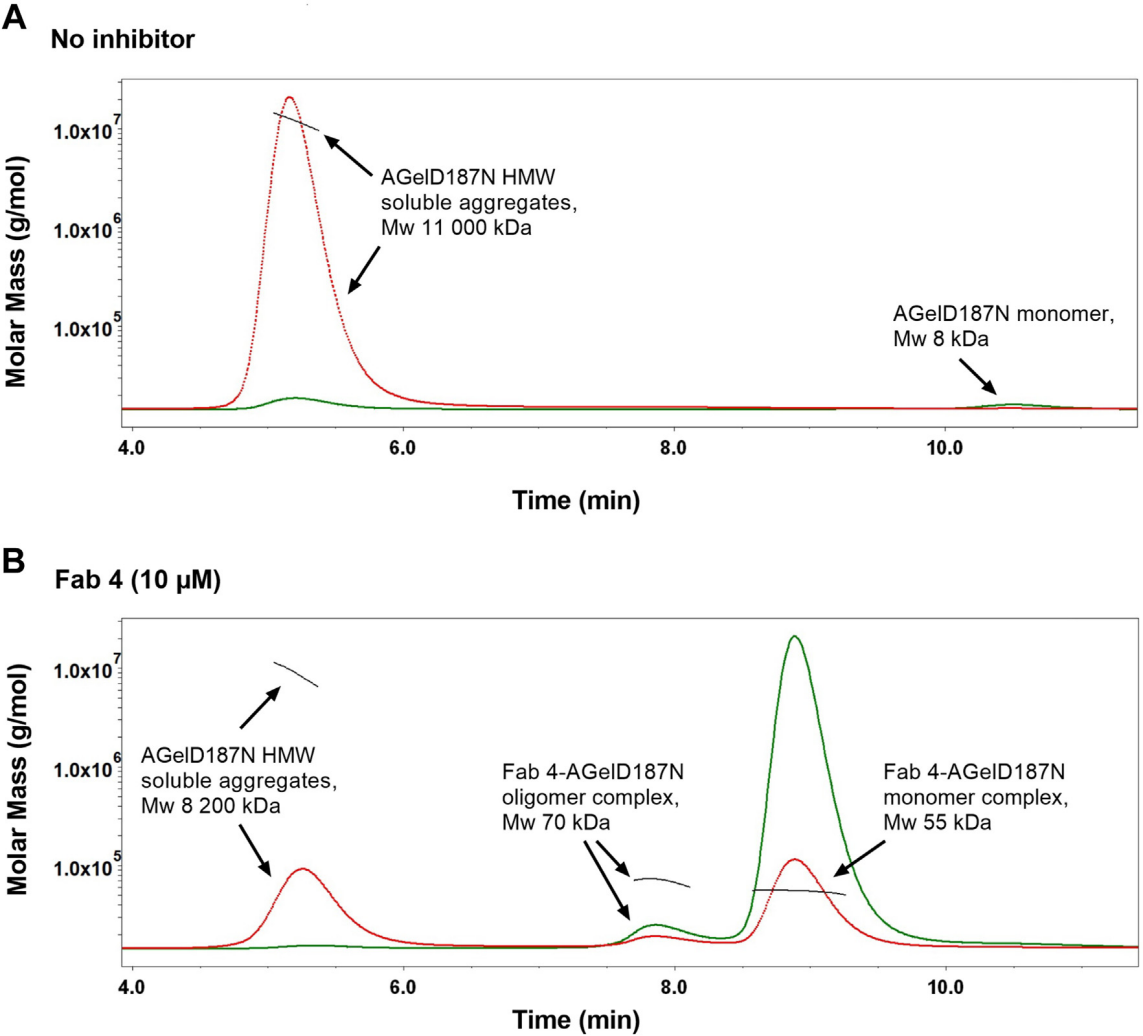
**SEC-MALS analysis of the assay supernatants after the seeded aggregation assay.***Green curve*: UV signal, *red curve*: light scattering (LS) signal. *A*, SEC-MALS analysis of the supernatant in the absence of Fabs. AGelD187N monomer and high-molecular-weight (HMW) soluble aggregate peaks were detected. *B*, SEC-MALS analysis of the supernatant in the presence of 10 μM Fab 4. The AGelD187N monomer peak was not detected. Fab 4-AGelD187N monomer and oligomer complex peaks were detected. The AGelD187N HMW soluble aggregate peak is reduced to 29% (LS peak area) of the original size. Fab, antigen-binding fragment; SEC-MALS, size-exclusion chromatography combined with multiangle light scattering.

The pathogenicity in protein misfolding diseases arises at least partly from the soluble oligomers generated in the process of aggregation (32, 33, 34). Interfering in the aggregation process can also lead to the formation of other, possibly off-pathway species, that can also be cytotoxic (35). If cross-β structures have not formed, ThT does not necessarily reveal these aggregated species (36). To confirm that the anti-AGelD187N Fabs neutralized the monomers efficiently and no off-pathway species were formed, SEC analysis coupled with monitoring through multiangle LS was performed on the assay supernatant of Fab 4 sample. The solubility of the amyloidogenic protein will define the quantity of free monomers in the supernatant after the aggregation reaction (37). Moreover, prefibrillar intermediates that are small enough to enter the column bed will appear in the void volume of the column (38, 39, 40). As expected, AGelD187N monomers and high-molecular weight (>10,000 kDa) soluble aggregates were detected in the sample without inhibitor (Fig. 5*A*). In the sample with Fab 4, no free monomers or low-molecular-weight soluble aggregates were detected after the aggregation reaction. Instead, a main peak of 55 kDa and a second, minor peak of 70 kDa were detected (Fig. 5*B*). As the molecular weights of the Fab 4 and AGelD187N monomers are 47 kDa and 8 kDa, respectively, these results clearly indicate that Fab 4 formed 1:1 complexes with AGelD187N monomers. The peak of 70 kDa could indicate the formation of a small number of complexes between Fab 4 and AGelD187N trimers. In addition to the formed antibody complexes, the high-molecular weight soluble aggregates were reduced to 29% when comparing LS peak areas. The above results were consistent with the kinetic curves that showed the blocking of aggregation in the presence of 10 μM Fab 4.

## Discussion

In this study, we have described the discovery of high-affinity antibody fragments that bind to different epitopes of monomeric AGelD187N, an aberrant amyloidogenic fragment of plasma gelsolin that causes hereditary gelsolin amyloidosis. We also have shown that the antibody fragments efficiently inhibited the aggregation of the disease-relevant full-length AGelD187N *in vitro*, although only one of the antibody fragments was known to bind to the previously proposed amyloidogenic region of AGelD187N.

Because of their high specificity and binding affinity, a wide range of antibodies have been generated against a variety of proteins and peptides involved in protein misfolding diseases (41). However, to the best of our knowledge, no antibodies have been reported that target the aberrant amyloidogenic peptide AGelD187N in Finnish gelsolin amyloidosis, although the pathophysiology of this systemic hereditary disease is straightforward and well understood, unlike in other protein misfolding diseases. Unlike most misfolding proteins, the AGelD187N monomer does not have an innate form in the body and is therefore a logical choice as a target to be neutralized. We aimed to identify high-affinity binders to monomeric AGelD187N that would stabilize the amyloidogenic fragment and inhibit pathological aggregation, providing an early intervention for the disease. Antibodies targeting the same antigen may elicit different responses, or even opposite reactions by binding to distinct molecular features or conformations. For the most-studied amyloidogenic proteins, several different linear and conformational epitopes have been suggested, some found only from monomers, some from aggregated species, and some from both (42, 43, 44, 45, 46). To efficiently inhibit monomer aggregation, it may or may not be necessary to target the amyloidogenic core of the protein (47, 48, 49, 50, 51). The amyloid core of patient-derived AGelD187N fibrils has not been defined by any structural analyses, but based on aggregation studies of short stretches of AGelD187N, an amyloidogenic region ^182^SFNNGDCFILD^192^ or even shorter ^188^CFILDL^193^ has been proposed (9, 52). We were interested in whether this sequence motif would need to be targeted for efficient inhibition of AGelD187N aggregation. Phage display is an efficient and fast way to target distinct antigen epitopes without the need for them to be immunogenic. In addition to full-length AGelD187N, three short regions of AGelD187N (^173^ATEVPVSWESFNNG^186^, ^181^ESFNNG**N**CFILD^192^, and ^227^GRARVHVSEEGTEPEA^242^) were used as selection antigens to generate antibody fragments that bind to different epitopes.

In summary, 18 scFv clones were isolated from an scFv phage display campaign, and five additional Fab clones were obtained after conversion of the enriched scFv pools to a library of Fabs that upon further phage selection rounds led to domain-pair optimization. For the aggregation inhibition assay, the best-affinity Fab from each selection antigen group was selected. The best and second-best aggregation inhibitors, Fabs 4 and 19, completely blocked amyloid formation at a stoichiometric ratio to full-length AGelD187N, although neither of them targeted the previously proposed amyloidogenic region of the peptide. In addition, the two other Fabs, Fab 21 binding to the proposed fibril-forming region and Fab 14 binding to an unknown region, clearly inhibited amyloid formation at a stoichiometric ratio. Surprisingly, Fab 14 appeared to induce amyloid formation at lower concentrations. However, this was unlikely, as the aggregation reaction was run to completion without an inhibitor, which was confirmed by measuring the free AGelD187N concentration remaining in the solution after the assay. The most probable explanation for this is that at lower doses, Fab interferes with the aggregation process in a way that changes the fibril morphology and consequently, ThT binding and fluorescence (53, 54, 55). Based on our study, we can conclude that the earlier proposed fibril-forming region of AGelD187N does not need to be targeted for efficient aggregation inhibition; conversely, some other regions might be more beneficial. By binding to an important flanking region adjacent to the fibril-forming region, the antibody fragments can stabilize AGelD187N or induce a conformational change that hinders aggregation (56). It is also possible that the earlier proposed fibril-forming region does not have a central role in the aggregation of AGelD187N as previously thought because the assumption is based solely on the aggregation propensities of isolated segments of AGelD187N. Now these antibodies provide a suitable tool to investigate the aggregation process of AGelD187N and the structural features of AGelD187N fibrils more thoroughly. By establishing a kinetic model, it can be identified, for example, which steps of the aggregation process the antibody fragments specifically target (37, 49). If a Fab fragment binds to an epitope that becomes buried inside the fibril, it does not have affinity for fibrils (48). Based on our study, all regions of the full-length AGelD187N are important in modulating its assembly; therefore, we recommend that all future studies related to the aggregation and aggregation inhibition of AGelD187N should be performed with the disease-relevant full-length amyloidogenic peptide containing amino acid residues 173 to 242.

Our studies clearly implicate that one tightly bound Fab fragment was needed to neutralize one AGelD187N peptide. At a stoichiometric ratio of 1:1 to AGelD187N peptide, the highest-affinity Fab fragment, Fab 4, blocked amyloid formation fully; at a 1:0.5 ratio, the maximum fluorescence intensity decreased by approximately 50%, and at a 1:0.25 ratio, by approximately 25%. For systemic delivery, the conversion of Fab fragments to the full IgG format would be needed to provide the required half-life for *in vivo*. One IgG molecule could then neutralize two AGelD187N peptides with its two Fab arms. However, considering that amyloid deposition in gelsolin amyloidosis is observed in multiple organs and tissues, the number of AGelD187N peptides emerging in the patient's body could be large. To neutralize enough AGelD187N monomers at reasonable antibody concentrations, affinity maturation of the developed antibody may be needed. Moreover, efficient clearance of the formed AGelD187N-antibody complexes might be needed in addition to binding interference. This could be achieved by engineering the antibody for efficient effector functions and pH-dependent antigen binding (57, 58, 59, 60). For instance, in a “sweeping antibody”, the constant and variable regions of the antibody are engineered for pH-dependency to enhance the neonatal Fc receptor (FcRn)-mediated uptake and recycling of the antibody-antigen complex, allowing the antibody to be recirculated while the antigen undergoes lysosomal degradation (61). As such, the anti-AGelD187N Fabs described here could be an attractive intravitreal treatment option against corneal amyloidosis, the earliest clinical finding in Finnish gelsolin amyloidosis (4). Several intravitreal therapies based on antibodies or antibody fragments have been approved and are under development (62). Another important application area could be the use of these anti-AGelD187N Fabs as diagnostic tools to detect circulating AGelD187N from patient blood and as noninvasive imaging probes to detect diseased tissues.

We conclude that this study provides the first demonstration of high-affinity binders and aggregation inhibitors to the aberrant amyloidogenic AGelD187 peptide, which causes gelsolin amyloidosis. The discovered antibody fragments bind to different epitopes in the disease-relevant full-length monomeric AGelD187N and efficiently inhibit its aggregation *in vitro*. The epitope-specific anti-AGelD187N Fabs can be exploited not only in fundamental research on gelsolin amyloidosis but also in developing currently lacking disease-modifying treatments and diagnostic tools for the disease. Due to the simple pathophysiology and accessibility of the amyloidogenic peptides, gelsolin amyloidosis could serve as an optimal model disease to test the still not fully validated amyloid hypothesis prevailing in the field of neurodegenerative diseases. Progress in alleviating tissue damage and clinical symptoms by inhibiting aggregation in gelsolin amyloidosis could encourage and guide the work among more complex protein misfolding diseases, which pose an enormous burden on innumerable individuals and society.

## Experimental procedures

### Peptides

#### Synthetic peptides

The selection antigens Ag2, Ag3, and Ag4 were supplied by GenScript Biotech. Screening antigens Ag1, Ag9, and the full-length AGelD187N 173 to 242 without biotinylation, used in the aggregation assay were supplied by Caslo Aps. The nomenclature used for the disease-relevant amyloidogenic peptide is based on the recommendations of the International Society of Amyloidosis nomenclature committee (63). The short name AGelD187N in the text refers to the full-length AGelD187N 173 to 242.

#### Production of recombinant Ag7

A synthetic gene coding for a 6His-tag, an Avi-Tag, and AGelD187N 173 to 242 (Genscript) was cloned using restriction enzymes HindIII and XbaI into expression vector pHAT. This plasmid was amplified in *E. coli* XL1-Blue cells (Agilent Technologies) and used to transform heat shock competent BL21/BirA+ cells (dept. Biochemistry, University of Cambridge, UK, similar to *E. coli* strain AVB101 from Avidity). The cells were recovered in super optimal broth with catabolite repression medium for 90 min at 37 °C and cultured on agar plates. Single colonies were used to seed 20 ml super broth medium (25 μg/ml chloramphenicol and 100 μg/ml ampicillin), which was incubated at 37 °C until *A*_600_ = 7.5 in a primary culture and further in a 500 ml culture to *A*_600_ = 0.8. Expression was induced with 400 μM IPTG in the presence of 50 mM biotin overnight at 30 °C. Cells were pelleted by centrifugation for 15 min at 6000*g* at 4 °C. The pellet was resuspended in PBS containing 10 mM imidazole and lysed using an Emulsiflex C3 homogenizer (Avestin). DNAse was added, and the suspension was incubated at room temperature (RT) for 15 min. The cell debris was centrifuged at 28,000*g* at 4 °C for 30 min. The supernatant was collected and Ag7 was purified by nickel-nitrilotriacetic acid (Ni-NTA) affinity chromatography and SEC (Superdex 10/200 Gl column) chromatography.

### Biopanning

Using a solid phase panning protocol, biotinylated peptide antigens were loaded to saturation on 400 μg streptavidin beads (Dynabeads M-280, Invitrogen) or epoxy beads (Dynabeads M270 Epoxy, Invitrogen) coated with neutravidin. The beads were incubated with the primary scFv library (10^12^ cfu) or a phage pool of scFv or Fabs (5·10^10^ cfu) from a previous panning round, in selection buffer (PBS containing 0.1% bovine serum albumin (BSA) and 0.05% Tween-20) at RT for 30 to 60 min. Using the solution phase protocol, the antigens (0.1 nM or 1 nM) and phage pools were preincubated at RT for 30 to 60 min, and subsequently mixed with streptavidin beads for 5 min. The bead-bound antigen–phage complexes were collected from the selection buffer using a bar magnet (DynaMag-2, Invitrogen). Beads were washed three times with selection buffer and once with PBS. Antibody phage elution, rescue by infection, production of new phage stocks, and monitoring of the panning process were performed as described previously (64). In brief, phages were eluted with trypsin and rescued by infecting midlog *E. coli* XL1-Blue cultures for phage propagation. Phage stocks were prepared the following day with PEG-NaCl precipitation, and the selection was repeated for four rounds with scFv phage libraries and further three rounds with Fab libraries. The progress of panning was monitored by dilution plating of infected cells from phage outputs and phage immunoassays. Biotinylated antigens (100 nM) were immobilized at RT for 30 min to streptavidin-coated streptavidin microplates (Kaivogen). The wells were washed four times before the addition of phage library (5 × 109 cfu/ml) at RT for 1 h followed by washing again and the addition of anti-phage-Europium-IgG (Eu-N1 αVCSM13, aE7A6G10 in tris-buffered saline with 1% BSA and 0.02% NaN_3_, 6.9 Eu/IgG, 225 ng/ml) at RT for 45 min. Finally, the Eu was enhanced with Delfia technology (PerkinElmer) and measured for time-resolved fluorescence using a Victor 1420 Multilabel Counter (Wallac).

### VL/VH shuffling

ScFv fragments were converted to Fab fragments with Golden Gate assembly and cloned with SfiI back to pEB32x phagemid for phage display. To this end, The VL and VH regions of enriched scFv pools were amplified by PCR (Phusion DNA polymerase, Thermo Fisher Scientific) from extracted pEB32x-scFv phagemid library DNA and human CL and CH1 genes from pLK06FT-Fab13 DNA (65) using primer pairs described previously (66). The resulting amplicons were purified with a PCR purification kit (Zymo Research), digested with LguI (Thermo Fisher Scientific) and repurified. The four DNA fragments with cohesive ends were ligated with T4 DNA ligase (Thermo Fisher Scientific). As the cohesive ends are unique, the ligation product yields Fab fragments with correct fragment orientations creating a bicistronic VL-CL and VH-CH1 construct. The product was further digested with DpnI (Thermo Fisher Scientific) to remove carryover caused by methylated pEB32x-scFv DNA template and KpnI (Thermo Fisher Scientific), which cuts scFvs at the linker region avoiding undesired amplification of scFvs in the following step. The enzymes were heat-inactivated, the Fab genes amplified with PCR (Phusion) using flanking primers and the correct size amplicons gel extracted from 1% agarose gel (Gel Extraction Kit, Thermo Fisher Scientific). The Fab fragments were digested with SfiI (Thermo Fisher Scientific), followed by purification (PCR purification kit, Zymo Research) and ligated (T4 DNA ligase, Thermo Fisher Scientific) to predigested pEB32x SfiI-vector fragment in 3:1 M ratio. The pEB32x vector containing the VL/VH-shuffled library was transformed into SS320 cells by electroporation (Bio-rad, Gene Pulser Xcell) with settings 2.5 kV, 200 Ω, and 25 μF. Following the pulse, the cells were recovered in super optimal broth with catabolite repression medium for 1 h at 37 °C and plated on selective agar plates (25 μg/ml chloramphenicol, 10 μg/ml tetracycline, and 0.5% w/v glucose). The preparation of phage pools, panning, expression, and screening was performed as described for scFv.

### Screening of isolated and soluble scFv and Fabs

The scFv encoding segments were subcloned into screening vector plK01 using SfiI restriction sites, expressed as monoclonal scFv-mCL fusion antibodies, purified by Ni-NTA affinity chromatography and SEC (Superdex 10/200 GL column) chromatography, and screened against peptide antigens with a time-resolved fluorometry-based immunoassay using an Eu-labeled anti-mCL antibody as the secondary antibody (64). Fabs were processed as described above for scFv, with the exception that Fabs were cloned into the pAK400 screening vector and detected with Eu-labeled anti-human IgG CH1 antibody 2A11 (Hytest).

### Epitope binning of isolated and soluble scFvs

Epitope specificities were determined by pairwise competition experiments using Octet RED96e or Octet RED384 instruments (ForteBio) at 20 °C in Octet kinetics buffer (PBS containing 0.1% BSA, 0.02% Tween-20, and 0.05% sodium azide). Streptavidin biosensors (Pall ForteBio) were loaded with a biotinylated antigen Ag 7 and subsequently dipped into solutions of two scFvs of equal concentration. This procedure was repeated for all combinations of scFv pairs, in both directions. An unchanged signal was interpreted as a shared epitope. An increase in signal upon exposure to the second scFv was interpreted as binding to an unoccupied epitope.

### Production of Fabs in CHO cells

#### Preparation of heavy chain plasmids

A synthetic VH-CH1-FLAG-gene carrying NotI and BamHI sites at the ends, and an NheI site before CH1 was ordered from GeneArt. This string was cloned into the NotI/BamHI-digested pSUP vector following NotI/NheI-digestion. The VH regions of scFv were amplified by PCR and cloned into pSUP using Gibson assembly (NEBuilder HiFi DNA Assembly Master Mix, NEB) to give plasmids for heavy chains.

#### Preparation of light chain plasmids

A synthetic VL-CL_Κ_-gene carrying NotI and BamHI sites at the ends and an AvaI-site before CL_Κ_ was ordered from GeneArt. This string was cloned into the NotI/BamHI-digested pSUP vector and NotI/AvaI-digested. The VL regions of scFv were amplified by PCR and cloned into pSUP using Gibson assembly to give plasmids for light chains.

#### Sequencing of cloned genes

All plasmids were sequenced using Sanger sequencing to confirm the sequence of the variable domains as well as that the cloning was done correctly.

#### Expression of Fabs in CHO cells and purification

Plasmids were purified from LB amp (Luria-Bertani broth containing ampicillin) cultures and transfected into 30 ml cultures of ExpiCHO cells (Gibco-Thermo Fisher Scientific) according to the manufacturer's instructions. ExpiFectamine CHO Enhancer and ExpiCHO Feed were added 19 h after transfection, and the cultures were incubated at 32 °C, 5% CO2 at 120 rpm for 11 days. The cultures were harvested by centrifugation. The secreted Fabs were purified using affinity chromatography CaptureSelect CH1-XL (Thermo Fisher Scientific) and SEC (Superdex 200 Increase 10,300 GL) on an ÄKTA Pure 25.

#### SDS-PAGE analysis

Fabs expressed in CHO cells were analyzed in reduced conditions on SDS-PAGE Mini-Protean TGX Precast gel (Bio-Rad). Precision Plus Dual Protein Standard (Bio-Rad) was used as the reference.

### Binding kinetics of scFvs and Fabs using biolayer interferometry

Binding kinetics of scFvs and Fabs to variants of AGelD187N were evaluated using Octet RED96e or Octet RED384 instruments (ForteBio) at 20 °C in Octet kinetics buffer. Streptavidin biosensors (Pall Forte Bio) were loaded with biotinylated antigens Ag1 or Ag9. Antibody fragments were added as analytes. Single-concentration KD values were determined using constant concentrations of antibody fragments: 200 nM for scFvs and 300 nM for Fabs. Multiple concentration kinetics of selected scFvs were determined using the parallel sensor kinetics method at seven concentrations between 3.25 nM and 200 nM. Curves were fitted and kinetic parameters were calculated using Octet analysis software (version 11, https://www.sartorius.com/en/products/biolayer-interferometry/octet-systems-software). Multiple concentration kinetics of selected Fabs were determined using the kinetic titration series method at six concentrations between 31 nM and 1 μM. Curves were fitted, and kinetic parameters were calculated using TraceDrawer software (Ridgeview Instruments, https://tracedrawer.com/).

### *In vitro* AGelD187N aggregation assay

The inhibitory effect of the developed Fabs on AGelD187N amyloid formation was assessed using a newly established method (31). Briefly, 8 kDa synthetic lyophilized AGelD187N peptide (Caslo) was dissolved in 6 M guanidine hydrochloride in PBS and sonicated in a water bath sonicator for half an hour to break up any aggregates or oligomers. The peptide solution was then centrifuged for 15 min at 20,000 rpm and loaded into a Superdex 75 Increase 10/300 Gl gel filtration column (GE HealthCare) preequilibrated with PBS to buffer exchange and remove any aggregated species. From the monomer peak, the main fractions were collected on ice, and UV-visible spectrophotometric measurement (DS-11, DeNovix) was used to determine the peptide concentration using an extinction coefficient of 12,490 M^−1^ cm^−1^. The monomerized peptide was diluted to 30 μM with PBS. Stock solutions of 500 μM ThT (Sigma-Aldrich) and 150 μM heparin (Mw = 17–19 kDa, Sigma-Aldrich) were prepared by dissolving the dry powders in PBS and filtering through a sterile 0.22 μm pore size polyethersulfone membrane filter (Corning) or a sterile 0.2 μm pore size Supor polyethersulfone syringe filter (Acrodisc, Pall), respectively. Freshly monomerized AGelD187N was further diluted with PBS and mixed with selected Fabs. The mixtures were incubated for 20 min at RT before adding heparin and ThT. The final concentrations in the reaction mixtures were 10 μM of AGelD187N, 2.5 μM, 5 μM or 10 μM of Fab, 10 μM heparin, and 50 μM ThT. The reaction mixtures were dispensed in a nonprotein binding half-area 96-well plate (ref. 3881, Corning) in three 100 μl replicates and each well was seeded separately with 0.6 μl preformed fibrils. The plate was sealed with sealing foil (LightCycler 480, Roche) as quickly as possible after seeding. Kinetic measurements were monitored at 37 °C in an EnVision 2105 multimode plate reader (PerkinElmer) by measuring ThT fluorescence at 485 nm (20 nm bandwidth) upon excitation at 440 nm (20 nm bandwidth) every 10 min for 40 h. Orbital shaking at 200 rpm (diameter 3 mm) for 15 s was performed before each measurement. Curve fitting and statistical analyses were performed using Prism 8 (GraphPad Software).

### Transmission electron microscopy

After the amyloid formation assay, the sealing foil was removed from the plate, and three replicates of each reaction mixture were pooled in 1.5 ml tubes. The carbon-coated copper grids (Formvar/carbon 200 mesh, prod. no. 01801, Ted Pella) were treated with glow discharge treatment (20 s), and an aliquot (4 μl) of each pooled well-mixed sample was placed on a separate grid. The samples were allowed to stand for 2 min on the grid before removing the excess solution with blotting paper. Each grid was washed twice with deionized water (4 μl) and negatively stained for 1 min with 2% uranyl acetate in water (4 μl). Excess uranyl acetate was removed with blotting paper. The samples were allowed to air dry for at least 5 min under the Petri dish cover. Transmission electron images were acquired using a JEM-1400Plus TEM with an acceleration voltage of 120 kV.

### Soluble aggregate analysis

After preparing the samples for electron microscopy, fibrils and insoluble aggregates were removed from the reaction mixtures by centrifuging the reaction mixtures at 20,000*g* at +4 °C for 1 h. The separated supernatants (100 μl) were analyzed for soluble aggregates and oligomers using SEC-MALS. Analytical SEC was performed using an AdvanceBio SEC 4.6 mm × 300 mm column (300 Å pore size, 2.7 μM particle size, Agilent Technologies) combined with an AdvanceBio SEC 4.6 mm × 50 mm guard column (300 Å pore size, 2.7 μM particle size, Agilent Technologies). Isocratic elution was performed using a mobile phase (150 mM sodium phosphate buffer, pH 7.0) flow rate of 0.35 ml/min. The column was connected to a bioinert HPLC system (1260 Infinity II, Agilent Technologies) sequentially with a diode array detector (DAD WR, 1260 Infinity II, Agilent Technologies), a MALS detector (miniDawn, Wyatt), and a refractive index (RI) detector (Optilab, Wyatt). The MALS detector used a laser source at 659 nm and three detectors at different angles. RI detection was performed at 658 nm. Output signals from the diode array detector, MALS, and RI detectors were imported into ASTRA software (Wyatt, wyatt.com) for data processing. A BSA standard (Wyatt) was used as an independent control for molecular weight.

## Data availability

All data are included within the article or supporting information.

## Supporting information

This article contains supporting information.

## Conflict of interest

This study has been conducted as a collaboration between Orion Pharma and the University of Turku. During the study, L. L. was employed by the University of Turku in a collaboration project funded by Orion Pharma, and P. H. and S. P. were employees of Orion Pharma. Currently, L. L. is employed by Orion Pharma, P. H. by Organon R&D Finland, and A. G. by Mobidiag. Orion Pharma provided funding but did not have any additional role in the study. Organon R&D Finland and Mobidiag had no role in the study. The remaining authors declare that they have no other conflicts of interest with the contents of this article.
