# Supplementary material for: Epitope-specific antibody fragments block aggregation of AGelD187N, an aberrant peptide in gelsolin amyloidosis
Source: J Biol Chem. 2024 Jun 27;300(8):107507. doi: 10.1016/j.jbc.2024.107507 (PMC11298591; doi:10.1016/j.jbc.2024.107507)
Supplement: Supporting Information [file mmc1.pdf]

Supporting information

Epitope-specific antibody fragments block aggregation of AGelD187N, an aberrant peptide in gelsolin amyloidosis

Laura Leimu[1,2]*, Patrik Holm[1,3,4], Anna Gąciarz[1,5], Oskar Haavisto[3], Stuart Prince[1,6], Ullamari Pesonen[2], Tuomas Huovinen[3] and Urpo Lamminmäki[3]*

[1]R&D, Orion Pharma, Orion Corporation, Turku, Finland; [2]Faculty of Medicine, Institute of Biomedicine, University of Turku, Turku, Finland; [3]Department of Life Technologies, University of Turku, Turku, Finland; [4]Organon R&D Finland, Turku, Finland; [5]Mobidiag, A Hologic Company, Espoo, Finland; [6]MediCity Research Laboratory, University of Turku, Turku, Finland

***Corresponding authors:**

Laura Leimu, Email. laura.leimu@orionpharma.com

Urpo Lamminmäki, Email. urplammi@utu.fi

Supplementary Tables and Figures

Table S1. Results from the third and fourth panning rounds of scFvs.

Table S2. Binding affinities and epitope specificities of selected anti-AGelD187N scFvs.

Table S3. Results from the third panning round of VL/VH-shuffled Fabs.

Table S4. Binding affinities of selected anti-AGelD187N Fabs.

Figure S1. SDS-PAGE analysis of the anti-AGelD187N Fabs secreted from CHO cultures.

Figure S2. Individual kinetic traces of amyloid formation of AGelD187N in the absence and presence of anti-AGelD187N Fabs.

Figure S3. Representative electron micrograph after AGelD187N aggregation assay performed in the presence of Fabs 19, 21, and 14.

Figure S4. The effect of Fab 4, Fab 19, and Fab 21 on the maximum fluorescence intensities in the AGelD187N aggregation assay.

Figure S5. The effect of Fab 14 on amyloid formation of AGelD187N.

Table S1. Results from the third and fourth panning rounds of scFvs. Panning results for each antigen are reported separately.

Antigen	Round	Avidin[c]	Panning protocol[d]	Enrichment by output colonies[e]	Enrichment by phage immunoreactivity (S/B)[f]	Primary screening hit rate (%)[g]	Unique clones[h]
Ag3	3	str	solution (1 nM)	464	1611	78	4/8
Ag3	4	ntr	solution (0.1 nM)	13	225	73	
Ag2	3	str	solid	27	424	97	4/7
Ag2	4	ntr	solution (0.1 nM)	7	936	57	
Ag4	3	str	solid	3200	411	39	4/8
Ag4	4	ntr	solution (1 nM)	2	280	30	
Ag7	3	str	solution (1 nM)	118	140	87	3/3
Ag7/3[a]	4	ntr	solution (0.1 nM)	1	n.d.	51	2/4
Ag7/2[b]	4	ntr	solution (0.1 nM)	1	n.d.	n.d.	n.d.

[a] In this case the first three rounds were panned against Ag7 and the fourth round against Ag3.
[b] In this case the first three rounds were panned against Ag7 and the fourth round against Ag2.
[c] Avidin coating used on magnetic bead: str = streptavidin, ntr = neutravidin.
[d] Panning protocol applied at each step. When panning on a solid phase, the avidin beads were loaded with an excess of antigen. The antigen-loaded beads were mixed with $5x10^{10}$ phage particles at RT and collected on a magnetic rack for elution. When panning in the solution phase, $5x10^{10}$ phage particles were mixed with 1 nM, 0.1 nM, or 0.01 nM antigen at room temperature. The formed phage-antigen complex was pulled down with naked avidin-coated magnetic beads before elution of phage. The concentration in brackets indicates the concentration of the antigen. For evaluation of enrichment, the number of colonies of infected cells growing on plates of actual selection was divided by that of non-antigen controls ([e]) and the ratio of the signal obtained with and without antigen in phage immunoassay was calculated ([f]).
[g] Hit rates indicate the portion of soluble, monoclonal scFvs showing > 5-fold binding to the selection antigen (as compared to non-antigen controls) in an immunoassay.
[h] Selected clones were sequenced, and their identities assessed concerning all CDRs.
n.d. = not determined

Table S2. Binding affinities and epitope specificities of selected anti-AGelD187N scFvs.

Phage display scFv clone	Later Fab clone	Selection antigen	K_D (nM)[a]	K_D (nM)[b]	Epitope specificity[c]
18E09	Fab 5	Ag3	-	96	unique
18G12	Fab 16	Ag3	-	235	unique
10D08	Fab 1	Ag2	4	-	unique
10E04	Fab 10	Ag2	-	-	-
16A05	Fab 3	Ag4	7	70	same as 16H06
16H06	Fab 4	Ag4	2	19	same as 16A05
12C02	Fab 2	Ag7	2	30	unique

[a] Measured with BLI (parallel sensor kinetics method) using Ag1.
[b] Measured with BLI (parallel sensor kinetics method) using Ag7.
[c] Epitope specificities were determined by pairwise binning experiments.
- = not detected

Table S3. Results from the third panning round of VL/VH-shuffled Fabs, derived from phage pools of scFv panning. Panning results for each antigen are reported separately.

ScFv phage pool used for construction of input pool	Panning antigen	Enrichment by output colonies[a]	Enrichment by phage immunoassay (S/B)[b]	Primary screening hit rate (%)[c]	Unique clones[d]
round 4, Ag3 and Ag7/3	Ag3	56	40	99	1/7
round 4, Ag2 and Ag7/2	Ag2	1	5	1	1/8
round 4, Ag4	Ag4	118	120	69	3/8
round 3, Ag7	Ag7	8	100	67	3/8

[a] The number of colonies of infected cells growing on plates of actual selection divided by that of non-antigen controls.
[b] Enrichment is given as signal-to-background ratio (S/B), as estimated by immunoreactivity in phage immunoassay.
[c]Hit rates indicate the portion of soluble, monoclonal Fabs showing > 5-fold binding to the selection antigen (as compared to non-antigen controls) in an immunoassay.
[d]Selected clones were sequenced, and their identities assessed concerning all CDRs.

Table S4. Binding affinities of selected anti-AGelD187N Fabs.

Fab clone	Selection antigen	K_D (nM)[a]	K_D (nM)[b]
Fab 3	Ag4	147	175
Fab 4	Ag4	66	105
Fab 7	Ag7	2199	1808
Fab 13	Ag4	79	132
Fab 14	Ag7	67	113
Fab 15	Ag4	123	129
Fab 19	Ag3	-	145
Fab 20	Ag4	34	71
Fab 21	Ag2	251	324
Fab 22	Ag4	71	116

[a] Measured with BLI (kinetic titration series method) using Ag1.
[b] Measured with BLI (kinetic titration series method) using Ag9.
- = not detected

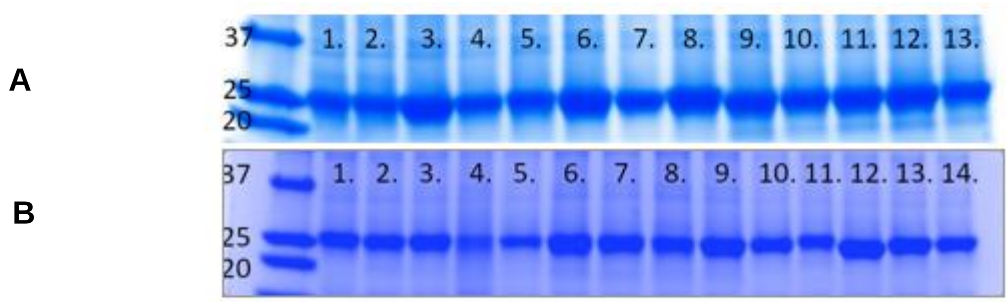

Figure S1. SDS-PAGE analysis of the anti-AGelD187N Fabs secreted from CHO cultures, reduced conditions. Fabs in order: (A) (1) Fab 1, (2) coFab 1, (3) Fab 2, (4) Fab 3, (5) Fab 4, (6) Fab 5, (7) Fab 6, (8) Fab 7, (9) Fab 8, (10) Fab 9, (11) Fab 10, (12) Fab 11. (B) (1) Fab 4, (2) Fab 12,(3) Fab 13, (4) Fab 14, (5) Fab 15, (6) Fab 05, (7) Fab 16, (8) Fab 17, (9) Fab 18, (10) Fab 19, (11) Fab 20, (12) Fab 21, (13) Fab 22, (14) Fab 23. Molecular weight markers were run in the first lane of each gel and the molecular weights of the reference bands are marked in kDa. Fab 4 and Fab 5 were loaded on both gels as cross-references for the staining and imaging of these two gels.

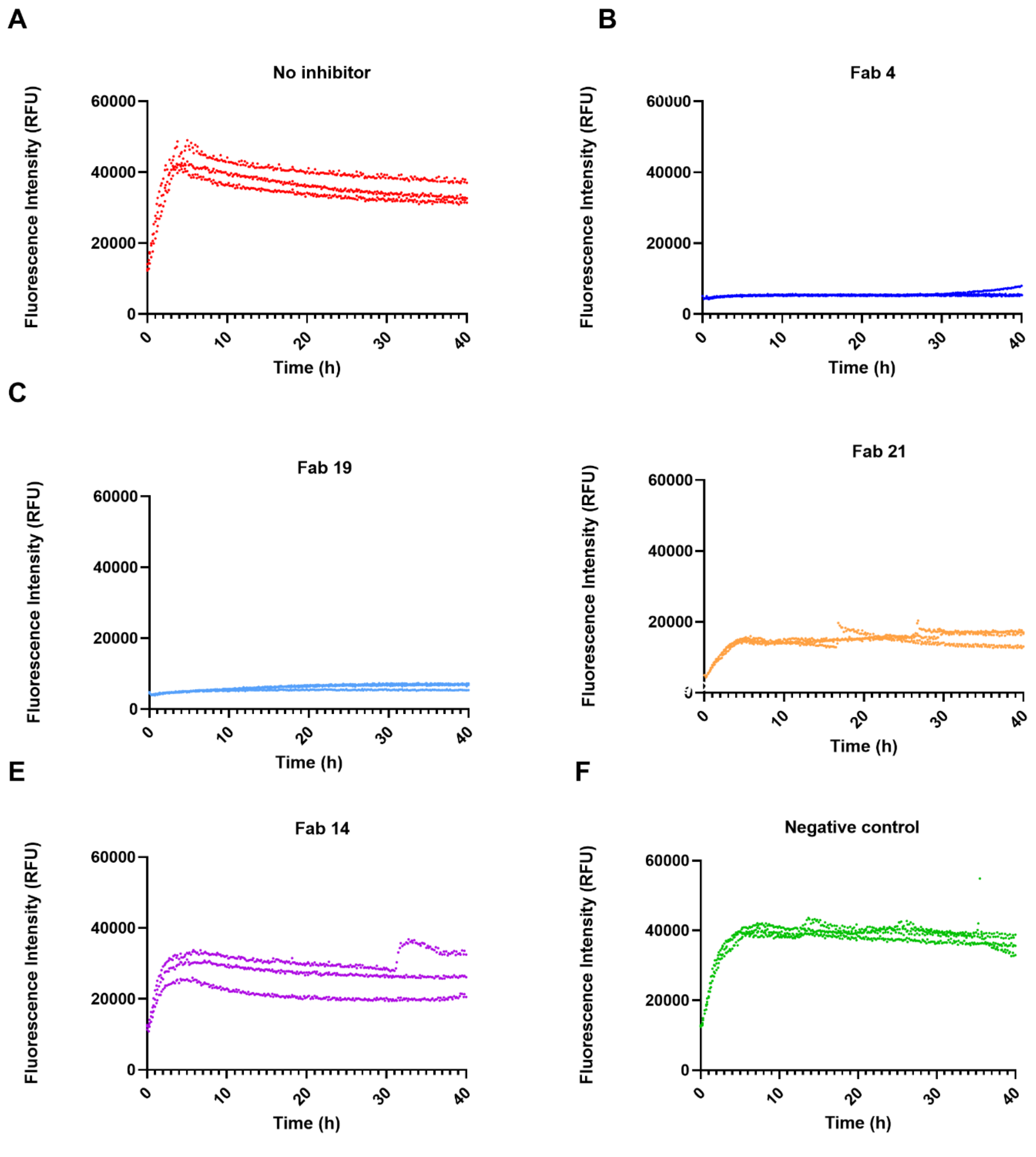

Figure S2. Individual kinetic traces of amyloid formation of AGelD187N (A) in the absence of Fabs, (B) in the presence of 10 µM Fab 4, (C) in the presence of 10 µM Fab 19, (D) in the presence of 10 µM Fab 21, (E) in the presence of 10 µM Fab 14, and (F) in the presence of 10 µM negative control, monitored continuously for 40 h by ThT fluorescence.

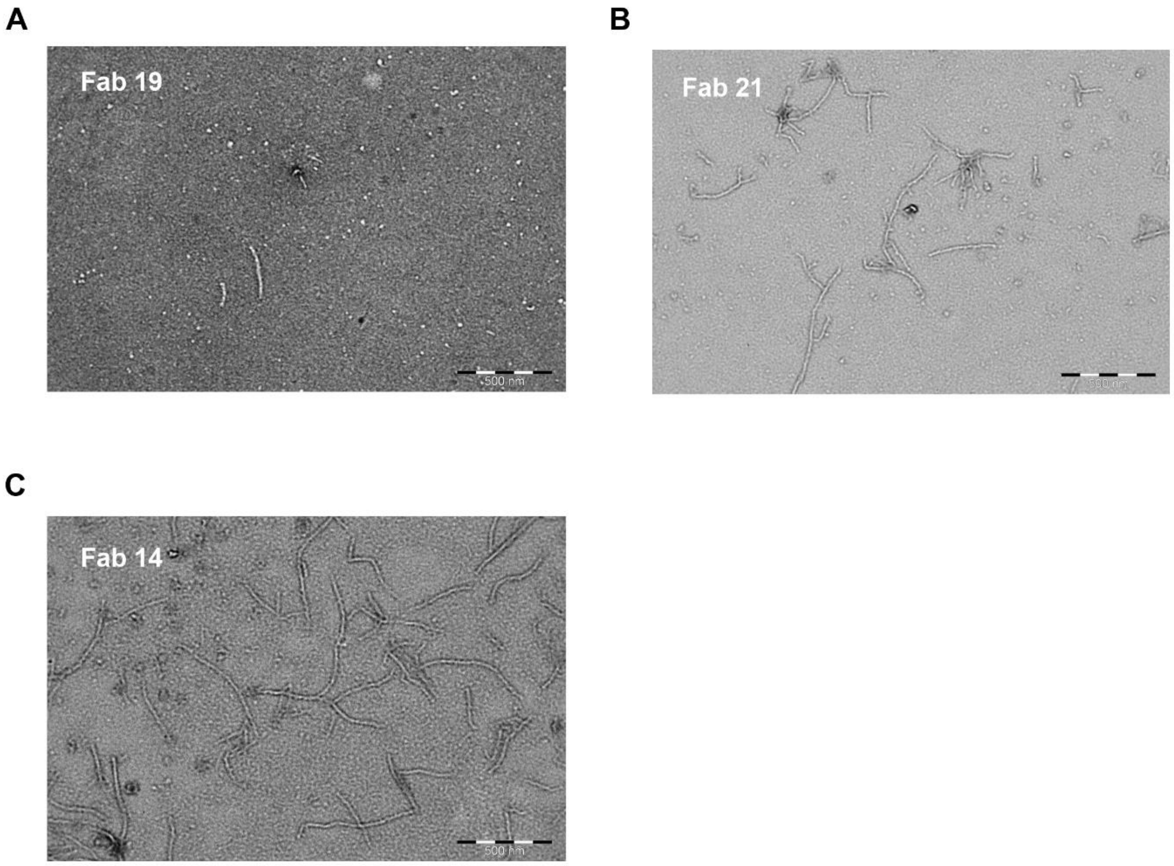

Figure S3. Representative electron micrograph after aggregation assay performed in the presence of 10 µM (A) Fab 19, (B) Fab 21, and (C) Fab 14. Scale bar 500 nm.

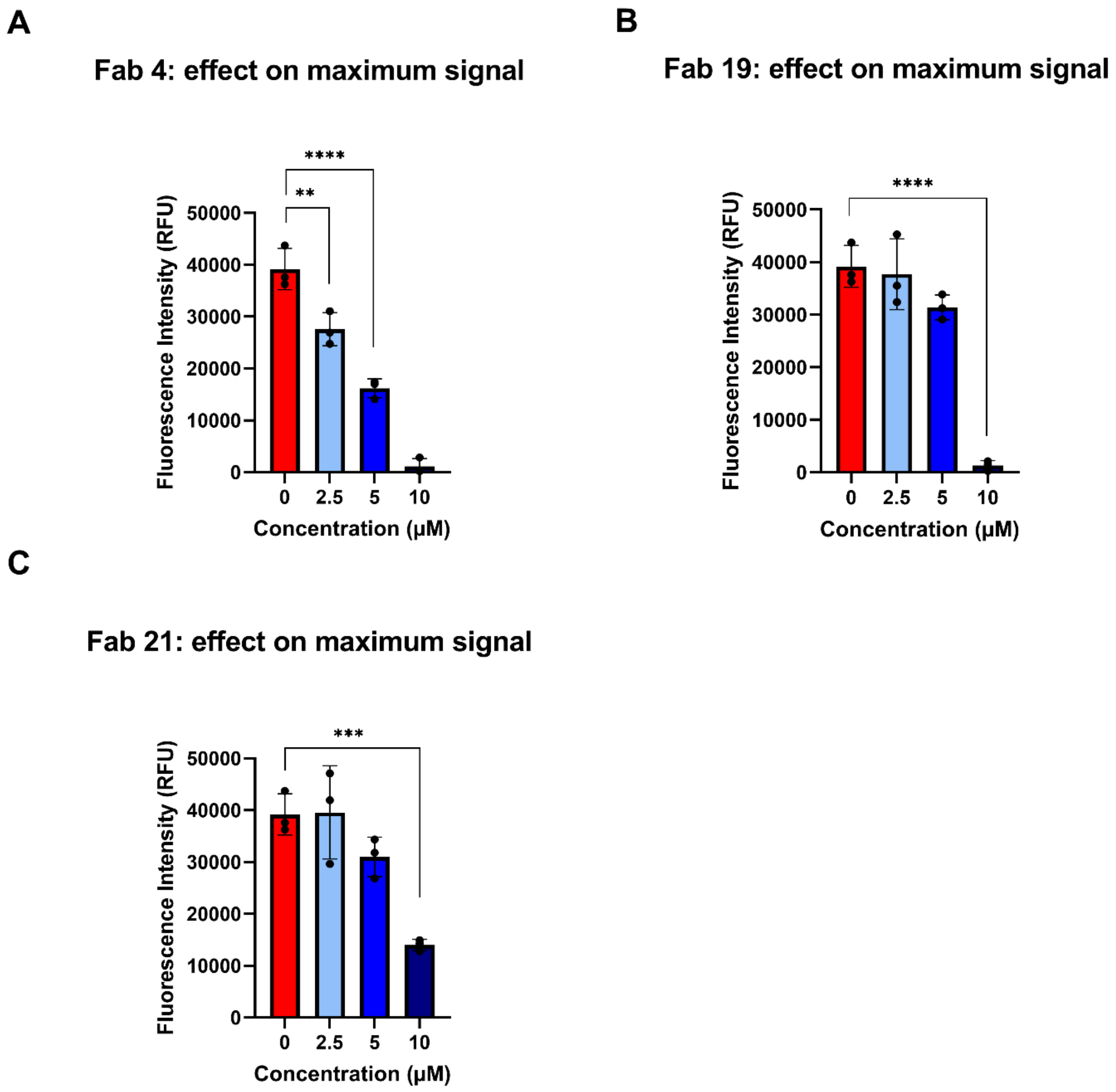

Figure S4. The effect of Fab 4, Fab 19, and Fab 21 on the maximum fluorescence intensities in the AGelD187N aggregation assay. (A) Maximum ThT fluorescence intensity in the presence of 0 µM, 2.5 µM, 5 µM, and 10 µM Fab 4. The data is baseline corrected and plotted as mean ± SD. The individual data points superimposed on each bar represent three replicate measurements. (B) Maximum ThT fluorescence intensity in the presence of 0 µM, 2.5 µM, 5 µM, and 10 µM Fab 19. (C) Maximum ThT fluorescence intensity in the presence of 0 µM, 2.5 µM, 5 µM, and 10 µM Fab 21. ****, $p < 0.0001$; ***, $p = 0.001$; **, $p = 0.021$ (one-way ANOVA test, followed by Dunnett´s multiple comparisons test).

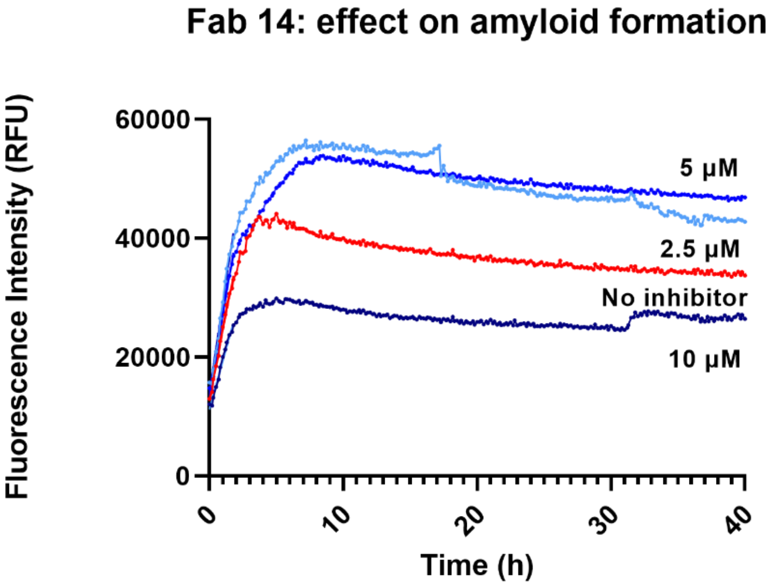

Figure S5. The effect of Fab 14 on amyloid formation of AGelD187N. Amyloid formation in the presence of 0 µM, 2.5 µM, 5 µM, and 10 µM Fab 14 monitored continuously for 40 h by ThT fluorescence. The mean curve of three replicate measurements is shown.
